# Disrupting the Climate Distress Cascade: Testing Community-Grounded Theories of Change Using Secondary Data From a Cross-Sectional Canadian Study

**DOI:** 10.1177/24705470261472655

**Published:** 2026-08-28

**Authors:** Aayush Sharma, Tim K. Takaro, Carmen H. Logie, Maliha Ibrahim, Kiffer G. Card

**Affiliations:** 1 Faculty of Health Sciences, 1763 Simon Fraser University, Burnaby, BC, Canada; 2 Factor-Inwentash Faculty of Social Work, 7938 University of Toronto, Toronto, ON, Canada; 3 483171 United Nations University Institute for Water, Environment, & Health, Richmond Hill, ON, Canada; 4 125319 Faculty of Behavioural Sciences, Yorkville University, Fredericton, NB, Canada

**Keywords:** climate change anxiety, psychological distress, resilience, social support, pro-environmental behaviour, belonging, climate perceptions, Canadian, structural equation modelling, community-grounded theories

## Abstract

**Background:**

Climate change is increasingly recognized as a source of significant psychological distress. How can we best minimize its mental health impacts? This study examines community-grounded theories of change by modelling how cognitive, emotional, and social processes shape climate-related psychological distress and adaptation.

**Methods:**

This cross-sectional study surveyed Canadian adults, measuring climate change perceptions (reality, causes, consequences, and psychological distance), climate worry, psychological distress, climate change anxiety, resilience, social support, belonging, and pro-environmental behaviour. Structural equation modelling (SEM) was used to test three theoretically informed models: (1) a cascade from climate perceptions to distress via worry and impairment, (2) a social-ecological model of support, resilience, and belonging, and action and (3) a combined model integrating cognitive, emotional, and social pathways.

**Results:**

Of 840 participants, 665 completed the survey. The mean sample age was 45·3 years (SD = 18·17). 70·3% identified as women, 18·3% as men, and 11·4% as gender diverse. Model 1 and Model 2 showed excellent fit. Model 3, which had a strong fit, showed that greater climate change perceptions predicted higher climate worry (β = 0·46, p <0·001), which led to greater impairment (β = 0·91, p <0·001), which led to distress (β = 0·32, p <0·001). Greater climate change perceptions had a buffering effect on impairment (β = -0·21, p < 0·001) and predicted distress (β = 0·55, p < 0·001). Stronger social networks predicted increased belonging (β = 0·85, p <0·001) and resilience (β = 0·61, p <0·001), each of which buffered psychological distress (resilience: β = -0·29, p <0·001; belonging: β = -0·06, p = 0·028). Resilience was a buffer against climate change perceptions (β = -0·43, p <0·001) and climate change worry (β = -0·28, p <0·001). Pro-environmental action predicted stronger climate change perceptions and climate worry (perceptions: β = 0·38, p <0·001; worry: β = 0·33, p <0·001).

**Conclusion:**

These findings support the value of community-based and psychologically informed approaches for minimizing climate-related psychological distress. Interventions that prioritize social support and emotional skills, together with accurate climate communication, may be critical for fostering mental health and collective resilience in a changing climate.

## Introduction

Climate change represents one of the greatest threats to human health and well-being in the 21^st^ century.^1^ A rapidly growing body of evidence highlights the profound mental health consequences associated with the climate crisis.^2,3^ Experiences of climate change, ranging from acute disasters to slow-onset disruptions, can trigger a broad spectrum of psychological responses, including worry, anxiety, grief, and trauma, which, in turn, affect daily functioning, life decisions, and overall well-being.^4^ These impacts manifest across diverse biopsychosocial domains: individuals may experience intrusive thoughts, sleep disturbances, diminished motivation, difficulty concentrating, and even changes in relationships, career choices, and reproductive planning, as seen in a recent survey of young Canadians.^5-8^ Understanding and mitigating these mental health burdens has become essential for individual quality of life, collective resilience, and adaptation in the face of accelerating climate threats, and for motivating action on emissions mitigation.

It is critical to distinguish among related, but conceptually distinct, constructs: climate change perception, worry, climate anxiety, and psychological distress. Accurate measurement of climate risk is foundational for adaptive behaviour and collective action.^9-11^ When individuals and communities possess a well-informed understanding of climate threats, debilitating impacts of complacency, inaction, or overwhelming fears can be contained.^2,12^ Climate worry, a natural and rational response to the seriousness of the crisis, can motivate engagement and preparedness.^13^ However, when worry becomes chronic or excessive, it may develop into climate anxiety, which can be debilitating, manifesting as intense rumination, panic, or avoidance.^5^ For example, research has demonstrated that higher climate change anxiety is associated with greater engagement in pro-environmental behaviour, a phenomenon we have called the “concerned steward effect”.^14^ However, this concerned steward effect can be attenuated at higher levels of distress, with effects such as eco-paralysis potentially stemming from limited returns on engagement or interpersonal challenges caused by engaging with other high-distress individuals.^15^ Eco-anxiety of this nature can impact social, academic, and occupational functioning, and, if unaddressed, escalate into psychological distress and restriction.^5,16,17^ This, in turn, has profound and inequitable consequences on people attempting to address the environmental crises, such as environmental advocates, activists, and researchers.^18-20^ Activist burnout is another related factor, defined as activists being forced to step back from activism, with the stress and pressure of their activism cited as a reason.^19^ Those suffering from activist burnout also deal with mental health consequences, including stress and depression.^19^

The need to mitigate distress, therefore, is not solely about symptom reduction but also to foster psychological, social, and civic conditions that enable people and communities to engage with distressing climate information, make adaptive decisions, and sustain collective responses to ongoing and future challenges, i.e. enhance individual and collective agency in the climate crisis.^21^ Recognizing this, a range of community-based and research-informed strategies have been developed to address climate anxiety and its sequelae (i.e, Climate cafés and community climate hubs).^22-28^ These interventions rely on the theory that climate perceptions can contribute to distress and that social support can help buffer this effect by promoting effective coping strategies.^29,30^ For interventions focusing on mobilizing climate action,^26,27^ psychological activation in response to climate change may be a normal, even positive, response to the climate crisis and provide an opportunity for individuals to channel their negative emotions into positive action.^31-33^ Other forms of coping strategies for climate change-related impacts exist, including emotional coping, often described as distancing and minimizing concern, and meaning-based coping, which involves finding positives in difficult situations or drawing on larger-level goals and beliefs to promote well-being.^34,35^ There have been limited empirical evaluations of these theories, either through program evaluations or observational research. This study provides novel contributions by assessing various theoretical pathways in the “climate cascade”, by examining pathways to distress, potential buffering effects, and the effects of pro-environmental behaviour, using individual and combined SEM models.

The present study aims to build a case for future longitudinal intervention research to understand the mechanisms by which these programs could be most effective by exploring inter-relationships between the cascading mental health consequences of climate change (i.e., climate perceptions, worry, anxiety/impairment, and distress) and the positive influences of social support and belonging, psychological resilience, and pro-environmental behaviour. Based on the literature characterized above, we hypothesize that such analyses will reveal complex inter-relationships: (a) climate perceptions will be related to worry and subsequently to impairment and distress; (b) social support will help to buffer the effects of perceptions on distress, by promoting climate action, belonging, and resilience; and (c) the climate cascade is associated with climate action.

## Methods

### Participants

This secondary data analysis utilizes data from a community-based participatory research project aimed at developing and validating measures of climate-related ecological distress and resilience. Information about the broader study is reported on the Mental Health and Climate Change Alliance website (https://mhcca.ca/publications). Summarizing this information, participants were individuals, aged 16+, residing in Canada, who were recruited through a combination of (a) paid and organic online advertisements, including via Instagram and Facebook, and (b) through organic outreach to the network and listserv of the Mental Health and Climate Change Alliance (https://www.mhcca.ca/), a non-profit organization engaged at the intersection of mental health and climate change with connections across organizations in Canada.

All participants accessed the survey via a secure online link, hosted on Qualtrics, and provided informed consent prior to participation. The survey comprised validated and study-developed measures capturing climate change perceptions, worry, psychological distress, climate-related impairment, social connection, resilience, belonging, and pro-environmental behaviour. Data was collected between July 2023 and April 2024. The study protocol was reviewed and approved by the Research Ethics Board at Simon Fraser University (REB#: 30000969).

### Data Collection and Preparation

To prepare the data for analysis, responses were processed and scored according to published instructions for each measure. The present study relied on scales previously validated by other research teams. Key details of the scales can be found in Table 1. Full details of the scales and their use in this study are provided in the Appendix (Appendix A).

**Table 1.** Climate Change-Related and Psychological Measure Scales Used in This Study

Construct	Scale	Scoring	Number of questions	Cronbach alpha	Sample question
Climate Change Perceptions	Valkengoed Climate Change Perceptions Scale (CCPS)^36^	7-point Likert Scale	5 questions	0·98. 0·97, 0·90, 0·93, 0·89 (for 5 subscales)	“I believe that climate change is real”
Climate Change Worry	Climate Change Worry Scale (CCWS)^37^	5-point Likert Scale	10 questions	0.95 (or 0.90/0.91)	“I worry about climate change more than other people.”
Climate Change Anxiety	Clayton and Karazsia Climate Change Anxiety Scale (CCAS)^5^	5-point Likert Scale	13 questions	0.92	“Thinking about climate change makes it difficult for me to sleep.”
Climate Perception and Worry	Six Americas Short SurveY (SASSY)^38^	5-point Likert Scale	4 questions	Not tested (test-retest reliability tested, with a Pearson Correlation Coefficient of 0.66)	“How important is the issue of climate change to you personally?”
Pro-Environmental Behaviour	Larson Pro-Environmental Behaviour Scale (PEBS)^39^	5-point Likert Scale	13 questions	0.64-0.84	“Thinking of your past behaviour, how frequently have you recycled paper, plastic and metal?”
Social Belonging	General Belongingness Scale (GBS)^40^	5-point Likert Scale	12 questions	0.80	“When I am with other people, I feel included.”
Social Network	Lubben Social Network Scale (LSNS-6)^41^	10-point Likert Scale	6 questions	0.83	“How many relatives do you see or hear from at least once a month?”
Brief Resilience	Brief Resilience Scale (BRS)^42^	5-point Likert Scale	6 questions	0.80-0.91	“I tend to bounce back quickly after hard times.”
Rugged Resilience	Rugged Resilience Measure (RRM)^43^	5-point Likert Scale	10 questions	0.87	“I believe in myself.”
Climate-Related Ecological Distress and Resilience	Climate-Related Ecological Distress and Resilience Scale (CEDAR)^44^	5-point Likert Scale	12 questions	0.861 for Distress and 0.769 for Resilience	“I feel motivated to take individual action towards protecting the environment”
Psychological Distress	Kessler Psychological Distress Scale (K10)^45,46^	5-point Likert Scale	10 questions	0.93	“During the last 30 days, about how often did you feel hopeless?”
Post-traumatic stress disorder (PTSD)	Post-Traumatic Stress Disorder Scale (PTSD-8)^47^	4-point Likert Scale	8 questions	0.83-0.85	“Recurrent thoughts or memories of the event”
Anxiety	Generalized Anxiety Disorder measure (GAD-7)^48^	4-point Likert Scale	7 questions	0.92	“Over the last two weeks, how often have you been bothered by the following problems?”; “Feeling nervous, anxious, or on edge.”
Depression	Patient Health Questionnaire (PHQ-9)^49^	4-point Likert Scale	9 questions	0.839	“Over the last two weeks, how often have you been bothered by the following problems?”; “Little interest or pleasure in doing things.”

Prior to analysis, all composite scale scores were calculated as described in Appendix A, and all variables included in the analysis were converted to numeric form. Only cases with complete data for all analytic variables were included in correlational and structural equation modelling analyses.

### Data Analysis

#### Descriptive Statistics

Descriptive statistics and Pearson correlations were computed for all study variables, with bivariate correlations and associated p-values estimated using the rcorr function from the Hmisc package (pairwise deletion for missingness).

#### Structural Equation Modelling

To test the study hypotheses, three structural equation models (SEMs) were specified and estimated using the lavaan package with maximum likelihood estimation and bootstrapped standard errors.

Model 1 (Climate Cascade) was designed to capture the potential sequential process by which cognitive appraisal of climate risk leads to worry,^50^ which may, in turn, increase both cognitive and functional impairment,^51^ ultimately culminating in greater psychological distress.^14^ Our study shows this theoretical cascade using climate change perceptions, represented by the Valkengoed Climate Change Perceptions Scale (CCPS),^36^ as the predictor of climate change worry, represented by the Climate Change Worry Scale (CCWS),^37^ which in turn predicted a latent impairment factor, which is modelled in this study as a factor underlying the cognitive and functional subscales of the Clayton and Karazsia Climate Change Anxiety Scale (CCAS).^5^ Impairment was then specified as a predictor of psychological distress, represented by the Kessler Psychological Distress Scale (K10).^45,46^ In this model, psychological distress (K10) was also regressed directly on climate change perceptions (CCPS) to test both direct and indirect effects. Residual covariance was specified between the two impairment subscales, allowing for shared variance not explained by the latent impairment factor, and potential overlap between climate worry and impairment on the directional path. The residual covariance specifications account for the possibility of direct links that are not fully captured by the hypothesized pathways.

Model 2 (Social Mitigators) was designed to capture the potential process by which one’s social network predicts belongingness,^52,53^ resilience,^54,55^ and climate action,^56,57^ in the context of climate change. The social network was modelled as a latent factor indicated by the Lubben “friends” and Lubben “relatives” subscales from the LSNS-6.^41^ Resilience is indicated as a latent factor, as measured by combining the Rugged Resilience Measure (RRM) and the Brief Resilience Scale (BRS).^42,43^ General belongingness, as measured by the GBS, was regressed on the social network latent factor to reflect theoretical and empirical support for the role of social integration in enhancing belonging.^40^ The resilience latent factor was regressed on the social network latent factor to capture the social origins of adaptive capacity. Pro-environmental behaviour, as measured by the PEBS, was regressed on the social network latent factor to test the hypothesis that individuals with stronger social ties are more likely to engage in climate action.^39^ Where modification indices suggested substantial unexplained variance among belonging, resilience, and pro-environmental behaviour, residual covariances were considered, consistent with theoretical links and prior research among these adaptive and behavioural outcomes.^58-60^

Model 3 (Combined Model) integrated both the climate cascade and social network models, providing a comprehensive test of direct, indirect, and buffering relationships among cognitive, emotional, social, and behavioural factors. In the final combined model, social network, resilience, and impairment were each modelled as latent factors, as seen in Models 1 and 2. Climate change worry (CCWS) was regressed on climate change perceptions (CCPS),^61^ the impairment latent factor was regressed on climate worry (CCWS) and climate change perceptions (CCPS),^10,62^ and psychological distress (K10) was regressed on both the impairment latent factor and climate change perceptions (CCPS) to test both mediation and direct effects, as supported by theoretical links in the literature.^63,64^ General belongingness (GBS) was regressed on the social network latent factor,^65^ the resilience latent factor was regressed on the social network latent factor, and climate change perceptions (CCPS) were then regressed on resilience,^66,67^ acknowledging that adaptive capacity is shaped by both social and cognitive resources.^68,69^ Climate change worry (CCWS) and climate change perceptions were regressed on pro-environmental action, modelling the joint influence of social and psychological factors on climate-related action.^70,71^ Psychological distress (K10) was also regressed on the resilience latent factor and belonging (GBS), testing whether these factors buffer the impact of climate-related risk and impairment on distress.^72,73^ Finally, climate change perceptions (CCPS) were regressed on the resilience latent factor, reflecting theoretical work on psychological coping and risk appraisal.^74^ At the same time, covariances were allowed among cognitive and functional impairment subscales (CCAS),^75^ between the social network latent factor and the impairment latent factor,^76^ between belonging (GBS) and the resilience latent factor,^77^ and between social network and pro-environmental behaviour (PEBS),^78^ consistent with theory and modification indices.

The Latent Variables used in this study were Impairment, Social Network, and Resilience, as these variables had validated subscales that captured their underlying constructs and did not weaken or destabilize the final model. Latent variables were tested using confirmatory factor analysis (CFA). Maximum Likelihood estimation with robust standard error values was used to account for non-normality,^79^ while Full Information Maximum Likelihood (FIML) was used to handle missing data during the CFA analysis.^80,81^ As all latent factors consisted of two variables, they were just-identified (df = 0). This meant that global fit indices (e.g., CFI, RMSEA, SRMR) were not interpretable as they would be suboptimal.^82,83^ Model validity is instead tested using statistical significance (p < 0.05) and factor loadings, with λ = 0.40-0.70 being considered moderate to strong.^84,85^ The impairment latent factor comprises the CCAS cognitive and functional subscales. Both factors loaded statistically significantly and strongly on the impairment latent factor (cognitive: λ = 0.887, p = .002; functional: λ = 0.891, p < .001). The resilience latent factor comprises the Brief Resilience Scale and the Rugged Resilience Measure. The factors both loaded strongly and significantly (BRS = λ = 0.865, p < .001; RRM λ = 0.865, p < .001). The social network latent factor comprises the Lubben Friends and Relatives subscales. Both factors loaded significantly (Friends = 0.717, p < .001; Relatives = 0.617, p < .001). Residual variances were positive across all models. These results support the use of these models and mean that the structural paths can be meaningfully interpreted. Other variables that could have been included as latent variables that had validated subscales included the PEBS and the CCPS. Both variables were tested as latent variables in the final model. The result was weaker, less stable models (e.g., CFI, TLI, RMSEA). Thus, these variables were included as composite scores instead.

Model fit was evaluated using chi-square, comparative fit index (CFI), Tucker-Lewis index (TLI), root mean square error of approximation (RMSEA), and standardized root mean square residual (SRMR). Acceptable model fit indices are x^2^ > 0 and with a p-value that is non-significant (>0·05), CFI > 0·90, TLI > 0·90, RMSEA < 0·08, SRMR < 0·10, based on prior research.^86^ Parameter estimates were obtained using nonparametric bootstrapping. All analyses were conducted in R, version 4·5·1. Complete case analysis was used for the SEM models. To test whether using FIML for missing data significantly altered the results, a sensitivity test was run for model 3. This modelling approach enabled a nuanced, theoretically informed investigation of how cognitive appraisals of climate risk, emotional and functional responses, and social connection interact to shape both psychological distress and pro-environmental adaptation.

## Results

### Demographics

A total of 840 participants were included in our analyses. Of the 840 participants, 665 (79·1%) finished the survey. Descriptive statistics are based on complete responses. Of those who completed the gender question, 70·3% of the sample identified as women (trans-inclusive), 18·3% as men (trans-inclusive), and 11·4% as gender diverse (agender, genderfluid, genderqueer, non-binary, or other). Of those who answered the respective questions, most participants were from British Columbia or Ontario (61·3%), aged 25-44 or 45-64 (65·9%), and had a household income exceeding $60,000 (58·2%). Full socio-demographic details can be found in Table 2.

**Table 2.** Socio-Demographic Characteristics of the Sample

Socio-demographic characteristic	Sub-category	N (%)
Gender	Woman (Trans-inclusive)	542 (70.3%)
	Men (Trans-inclusive)	141 (18.3%)
	Gender Diverse (Agender, Genderfluid, Genderqueer, Other Term, Non-Binary)	88 (11.4%)
Age	16-24	123 (15.0%)
	25-44	299 (36.5%)
	45-64	241 (29.4%)
	65 years or older	156 (19.0%)
Income	Less than $30,000	145 (19.4%)
	$30,000-$59,999	167 (22.4%)
	$60,000-$89,999	147 (19.7%)
	$90,000 or more	287 (38.5%)
Province/Territory	Alberta	111 (13.7%)
	British Columbia	254 (31.3%)
	Manitoba	35 (4.3%)
	New Brunswick	16 (2.0%)
	Newfoundland and Labrador	10 (1.2%)
	Northwest Territories	2 (0.2%)
	Nova Scotia	55 (6.8%)
	Ontario	243 (30.0%)
	Prince Edward Island	9 (1.1%)
	Quebec	47 (5.8%)
	Saskatchewan	27 (3.3%)
	Yukon	2 (0.2%)

*Note. Sample percentages are based on complete and eligible responses.

### Correlations

Pearson correlation analyses were conducted to examine the relationships among the primary study variables. Pairwise deletion was used to account for missing data.

All statistically significant correlations (p < 0·05) are reported in Table 3. The strength and direction of the associations were consistent with theoretical expectations: higher resilience and belonging were associated with lower levels of distress, worry, and impairment. Conversely, higher scores on PTSD, anxiety, depression, and climate change worry were strongly intercorrelated, as were climate impairment subscales. The CEDAR scale was also tested for correlations with the selected scales, with the results shared in the Appendix (Appendix B). Full results of the correlation analyses can be found in the Appendix (Appendix C).

**Table 3.** Pearson Correlations Among Study Variables

Variable	1	2	3	4	5	6	7	8	9	10	11
1. LSNS-6 friends score	1	0·44***	0·55***	0·35***	0·32***	0·14***	-0·17***	-0·06	-0·06	-0·11**	-0·07
2. LSNS-6 relatives score	0·44***	1	0·54***	0·33***	0·34***	0·10*	-0·28***	-0·15***	-0·17***	-0·20***	-0·19***
3. GBS	0·55***	0·54***	1	0·62***	0·58***	0·06	-0·47***	-0·28***	-0·24***	-0·33***	-0·33***
4. RRM	0·35***	0·33***	0·62***	1	0·75***	0·10*	-0·57***	-0·35***	-0·33***	-0·37***	-0·31***
5. BRS	0·32***	0·34***	0·58***	0·75***	1	0·02	-0·58***	-0·38***	-0·35***	-0·39***	-0·33***
6. PEBS	0·14***	0·10*	0·06	0·10*	0·02	1	0·27***	0·45***	0·34***	0·37***	0·37***
7. K10	-0·17***	-0·28***	-0·47***	-0·57***	-0·58***	0·27***	1	0·78***	0·82***	0·66***	0·59***
8. CCWS	-0·06	-0·15***	-0·28***	-0·35***	-0·38***	0·45***	0·78***	1	0·68***	0·74***	0·70***
9. CCPS	-0·06	-0·17***	-0·24***	-0·33***	-0·35***	0·34***	0·82***	0·68***	1	0·41***	0·38***
10. CCAS cognitive	-0·11**	-0·20***	-0·33***	-0·37***	-0·39***	0·37***	0·66***	0·74***	0·41***	1	0·79***
11. CCAS functional	-0·07	-0·19***	-0·33***	-0·31***	-0·33***	0·37***	0·59***	0·70***	0·38***	0·79***	1

*Note*. r values shown. *p < 0·05, **p < 0·01, ***p < 0·001 (uncorrected). All p-values for significant correlations are < 0·05 unless otherwise noted.

*Abbreviations*: LSNS: Lubben Social Network Scale; GBS: General Belongingness Scale; RRM: Rugged Resilience Measure; BRS: Brief Resilience Scale; PEBS: Larson Pro-Environmental Behaviour Scale; K10: Kessler Psychological Distress Scale; CCWS: Climate Change Worry Scale; CCPS: Valkengoed Climate Change Perceptions Scale; CCAS: Clayton and Karazsia Climate Change Anxiety Scale.

### Model 1 (Climate Cascade)

#### Model Fit

A structural equation model was estimated to evaluate a cascade pathway linking climate change perceptions (CCPS), climate change worry (CCWS), climate-related impairment (a latent variable comprising the cognitive and functional subscales of the CCAS), and psychological distress (as measured by the K10). Model fit indices indicated excellent fit: χ^2^(2) = 4·47, p = 0·11; CFI = 0·999; TLI = 0·995; RMSEA = 0·044 (90% CI [0·00, 0·10]); SRMR = .004. These results suggest that the model fits the observed data closely. The model explained substantial proportions of variance in all outcomes: cognitive impairment (R^2^ = 0·82), functional impairment (R^2^ = 0·71), climate change worry (R^2^ = 0·45), impairment latent variable (R^2^ = 0·64), and psychological distress (R^2^ = 0·82).

#### Path Coefficients

All paths were estimated using bootstrapped standard errors (based on 1,000 draws). Table 4 presents standardized path coefficients, standard errors, z-values, and p-values. Greater climate change perceptions were strongly associated with higher climate change worry (β = 0·67, p <0·001). In turn, climate worry predicted greater climate-related impairment (β = 0·65, p <0·001), modelled as a latent factor defined by cognitive (λ = 0·90) and functional (λ = 0·84) impairment subscales. Both greater impairment (β = 0·45, p <0·001) and climate change perceptions (β = 0·62, p <0·001) independently predicted higher psychological distress, indicating both direct and indirect effects. A path diagram for Model 1 is provided in the Appendix (Appendix D).

**Table 4.** Standardized Path Coefficients for the Perceptions–Worry–Impairment–Distress Model

Path	β (Std. All)	SE	z	p
**Direct Effects**				
Climate worry ← Perceptions	0·67	.05	23·54	<.001
Impairment ← Climate worry	0·65	.02	21·36	<.001
Distress ← Impairment	0·45	.07	16·92	<.001
Distress ← Perceptions	0·62	.05	30·93	<.001
**Factor Loadings**				
Impairment: Cognitive subscale	0·90	–	–	–
Impairment: Functional subscale	0·84	–	–	–
**Variance Explained (R** ^**2**^ **)**				
Cognitive impairment	0·82	​	​	​
Functional impairment	0·71	​	​	​
Climate worry	0·45	​	​	​
Impairment	0·64	​	​	​
Distress	0·82	​	​	​

*Note.* N = 635.

### Model 2 (Social Mitigators)

#### Model Fit

A structural equation model was estimated to examine how social network support (modelled as a latent factor with Lubben friends and Lubben relatives scores (LSNS-6)) predicts general belonging (GBS), resilience (latent factor: rugged (RRM) and brief resilience (BRS)), and pro-environmental behaviour (PEBS). The model demonstrated excellent fit: χ^2^(5) = 7·95, p = 0·16; CFI = 0·998; TLI = 0·993; RMSEA = 0·032 (90% CI [0·00, 0·07]); SRMR = 0·015. These indices suggest the hypothesized pathways provide a very good representation of the observed data. The model explained substantial variance in belonging (R^2^ = 0·68), resilience (R^2^ = 0·35), and the Lubben (Lubben Friends: R^2^ = 0·47; Lubben Relatives: R^2^ = 0·44) and resilience subscales (RRM: R^2^ = 0·83; BRS: R^2^ = 0·66). A low proportion of variance in pro-environmental behaviour was explained (R^2^ = 0·04).

#### Path Coefficients

Standardized path coefficients, standard errors, z-values, and p-values are provided in Table 5 (all estimated using bootstrapped standard errors, 1,000 draws). A stronger social network predicted higher general belonging (β = 0·82, p <0·001), higher resilience (β = 0·59, p <0·001), and greater pro-environmental behaviour (β = 0·20, p <0·001). Social network also strongly loaded onto both Lubben subscales (friends: λ = 0·68; relatives: λ = 0·66), and resilience was well-captured by both rugged (λ = 0·91) and brief (λ = 0·81) resilience subscales. A path diagram for Model 2 is shared in the Appendix (Appendix E).

**Table 5.** Standardized Path Coefficients for the Support–Belonging–Resilience–Action Model

Path	β (Std. All)	SE	z	p
**Direct Effects**				
Belonging ← Social network	0·82	.14	12·14	<.001
Resilience ← Social network	0·59	.10	9·12	<.001
Pro-environmental behavior ← Social network	0·20	.13	3·57	<.001
**Factor Loadings**				
Social network: Lubben friends	0·68	–	–	–
Social network: Lubben relatives	0·66	–	–	–
Resilience: Rugged resilience	0·91	–	–	–
Resilience: Brief resilience	0·81	–	–	–
**Variance Explained (R** ^**2**^ **)**				
Belonging	0·68	​	​	​
Resilience	0·35	​	​	​
Pro-environmental behaviour	0·04	​	​	​
Lubben’s friends’ score	0·47	​	​	​
Lubben’s relatives’ score	0·44	​	​	​
Rugged resilience score	0·83	​	​	​
Brief resilience score	0·66	​	​	​

*Note.* N = 569.

### Model 3 (Combined Model)

#### Model Fit

A structural equation model was specified to test the integrated effects of climate change perceptions, climate worry, climate-related impairment, social support, resilience, belonging, and pro-environmental action on psychological distress. The model included three latent variables: social network (LSNS-6 friends and relatives subscales, resilience (RRM and BRS), and impairment (CCAS cognitive and functional subscales). Model fit indices indicated fair fit to the data: χ^2^(32) = 67·45, p < 0·001; CFI = 0·991; TLI = 0·984; RMSEA = 0·046 (90% CI [0·03, 0·06]); SRMR = 0·035. Furthermore, the model accounted for large proportions of variance in most constructs: distress (R^2^ = 0·89), climate worry (R^2^ = 0·58), impairment (R^2^ = 0·68), belonging (R^2^ = 0·71), resilience (R^2^ = 0·38), but a small proportion of variance in climate change perceptions (R^2^ = 0·29). We also tested the latent variables for this model. Discriminant validity was assessed using the Fornell-Larcker and HTMT tests. For the Fornell-Larcker test, the sqrt(AVE) for the latent variables exceeds the correlations with any other variable,^87^ while the HTMT scores are below the required 0.85 threshold (range = 0.219-0.610),^88^ indicating that discriminant validity is upheld. Multicollinearity was assessed and cleared, with VIF scores below the 3.3 threshold (range = 1.27-1.91).^89,90^ Internal consistency and convergent validity were measured using Composite Reliability scores and Average Variance Extracted (AVE), respectively. These scores were strong for the resilience and impairment variables, comfortably meeting the thresholds of 0.6-0.7 for Composite Reliability and 0.5 for AVE.^91^ However, the convergent validity for the social network variable is below the 0.5 threshold at 0.442, while the internal consistency was at the lower end of the acceptable spectrum (0.612).^91^ As a result, we assessed the literature for studies that used the Lubben Social Network variables and examined how they applied them. Previous studies have used the Lubben Social Network as a sum total of the “Friends” and “Relatives” subscales.^92,93^ As a result, we conducted a sensitivity analysis to find whether the results would meaningfully change with a total Lubben score rather than a latent variable. However, the results were not meaningfully different, with the model including the latent Social Network variable performing marginally better on most metrics and significantly better in terms of variance explained. Another sensitivity analysis was conducted to assess whether using FIML to handle missing data yielded significantly different results. Our analysis showed that, although FIML recovered cases, it did not yield significantly different results. Full results of both sensitivity analyses are available in the Appendix (Appendix F and Appendix G).

#### Path Coefficients

**Figure 1.**
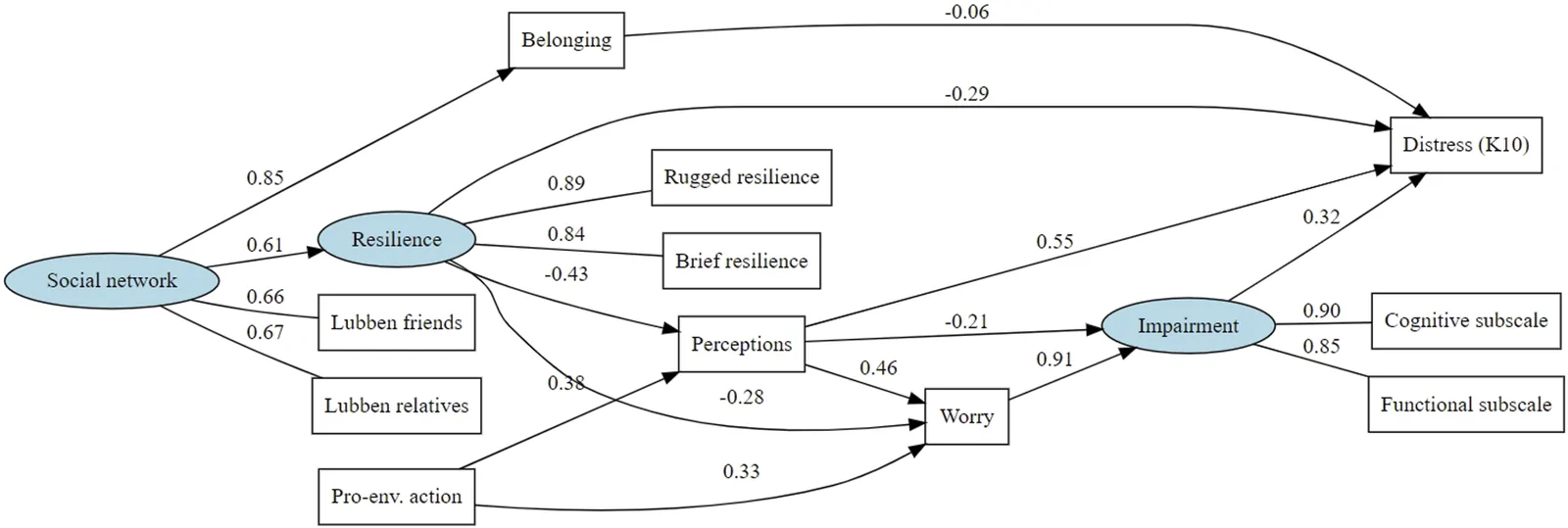
Path diagram for model 3 (combined model). *Note*. Values shown are the standardized path coefficients (β) for the SEM analysis.

All structural path estimates are fully standardized and based on bootstrapped standard errors. See Table 6 for details. Covariance results can be found in the appendix (Appendix H). In summary, climate change perceptions significantly predicted climate change worry (β = 0.46, p <0·001), which, in turn, strongly predicted climate-related impairment (β = 0·91, p < 0·001, latent). A direct path from climate change perceptions to impairment also showed a significant association, although the effect was reversed (β = -0·21, p <0·001). Both impairment (β = 0·32, p <0·001) and perceptions (β = 0·55, p < 0·001) were independent, significant predictors of distress. Social network robustly predicted general belonging (β = 0·85, p <0·001) and resilience (β = 0·61, p < 0·001). Resilience was also independently associated with lower distress (β = –0·29, p <0·001), climate change perceptions (β = -0·43, p <0·001), and climate change worry (β = -0·28, p <0·001), indicating negative associations. General belongingness was also associated with lower distress (β = –0·06, p = 0.028). Pro-environmental behaviour was a statistically significant predictor of climate change perceptions (β = 0·38, p <0·001) and climate change worry (β = 0·33, p <0·001). A path diagram with factor loadings for Model 3 is shared here (Figure 1).

**Table 6.** Standardized Path Coefficients for the Final Combined Social–Climate Cascade Model

Path	β (Std. All)	SE	z	p
Cascade Pathways				
Worry ← Perceptions	0.46	.06	12.66	<.001
Impairment ← Worry	0·91	.03	19·19	<.001
Impairment ← Perceptions	-0·21	.03	-5.33	<.001
Distress ← Impairment	0·32	.07	12·17	<.001
Distress ← Perceptions	0·55	.05	26·54	<.001
Social/Resilience Pathways				
Belonging ← Social network	0·85	.17	10·77	<.001
Resilience ← Social network	0·61	.11	8·13	<.001
Perceptions ← Resilience	–0·43	.04	–8·48	<.001
Worry ← Resilience	-0.28	.05	-7.44	<.001
Distress ← Resilience	–0·29	.06	–9·74	<.001
Distress ← Belonging	–0·06	.04	–2·20	.028
Action Pathways				
Perceptions ← Pro-env. action	0·38	.02	9·47	<.001
Worry ← Pro-env. action	0·33	·03	9·17	<.001

*Note.* N = 528.

## Discussion

### Primary Findings

This study examined the complex hypothesized pathways linking climate change perceptions, emotional responses, psychological distress, social connection, resilience, and pro-environmental behaviour among Canadian adults. Using a series of structural equation models, we found that climate change perceptions robustly predicted climate worry, which in turn was associated with increased impairment and, ultimately, heightened psychological distress (Model 1). Climate perceptions also directly predicted impairment and distress, having a buffering role on impairment while having a regular effect on distress. Importantly, while accurate perceptions and moderate levels of worry can be adaptive and motivating, both climate worry and distress were clearly linked to functional impairment, echoing concerns in the literature about the disabling effects of untreated eco-anxiety.^2,13,94^

Consistent with previous research, our analyses indicated that resilience and social support play a crucial role in mitigating the impacts of psychosocial stressors.^55,95-97^ Social network support predicted greater feelings of belonging and higher resilience, both of which were associated with lower psychological distress. These findings reinforce a growing body of evidence suggesting that supportive relationships and a sense of community are essential not only for buffering the psychological impacts of climate change but also for enabling adaptive coping and constructive engagement.^29,76,98^ The strong link between social network support and belonging is consistent with classic theories of social connection as a fundamental human need,^99^ and with contemporary models of community resilience.^100,101^ One’s sense of community is also key to ensuring collective action is effective.^102^ Resilience and belongingness also independently contributed to reduced distress, even after accounting for climate perceptions and impairment. These findings suggest that interventions aimed at reducing eco-anxiety, fostering resilience and a sense of belonging (e.g., climate cafés, peer support programs, citizen assemblies, and community organizing) can play a crucial role in promoting climate mental health and adaptation.^103^

In our study, pro-environmental behaviours predicted climate change perceptions and increased climate worry. The effect of social network on pro-environmental behaviour was significant in Model 2, whereas the two variables showed significant covariance in Model 3. This nuance suggests that the theoretical pathway from action to climate worry was not straightforward. Although pro-environmental, socially and community-sanctioned, cohesive climate action may strengthen one’s perceptions of climate change, it can also increase climate worry, which may align with prior research on the concerned steward effect.^14^ Previous research has shown that while impairment can drive engagement in climate action, excessive distress may actually inhibit action and engagement.^14,15^ Previous studies have shown that training and engaging in pro-environmental behaviours could help reduce climate worry, a finding that contrasts with this study’s findings.^104,105^

Furthermore, our findings that climate perceptions can shape distress and impairment highlight the need for balanced, supportive approaches to climate risk communication and public engagement.^106-108^ With this, it is important to note that the literature on whether general anxiety and eco-anxiety are distinct concepts is contested; some researchers have shown that the two concepts are associated with each other and may have potential overlap,^109,110^ while others argue that the symptoms and adaptive nature of eco-anxiety differ from those of general anxiety.^109,111-113^ Recent studies suggest that while the two concepts are correlated, they are not the same, with evidence showing that eco-anxious people do not have high levels of general anxiety.^114^

Additionally, it is important to note that there are well-documented uncertainties in climate health impact projections. For example, although researchers can, in most scenarios, estimate the direction and approximate the order of magnitude of impacts, this is not always the case.^115,116^ These uncertainties stem from multiple sources, including limited knowledge, potential confounding variables, complex relationships, and differing models or projections.^116,117^

### Implications

These results have important implications for practice and intervention. As our study found a theoretical pathway from climate risk perceptions to increased climate worry and general distress, and direct paths from perceptions to impairment and distress, it is essential to support individuals in developing both cognitive and emotional skills for managing climate information, including skills for emotion regulation, meaning-making, and perspective-taking, which may help improve their self-efficacy and mitigate the risk of chronic distress and eco-paralysis.^118^ Our study also found that social support improved resilience and a sense of belonging, which then both reduced distress. Thus, community-based interventions that strengthen social networks and foster a sense of belonging may serve as scalable, low-barrier means to promote resilience and adaptive coping.^119^ Efforts to support climate action should also acknowledge the psychological barriers posed by excessive worry, as shown in Model 1, and distress and incorporate components that promote agency, hope, and efficacy.^120,121^ Importantly, our findings suggest that supporting group-based and community-level pro-environmental behaviours may require strategies beyond simply reducing distress or increasing resilience at the individual level. Future interventions should explore how group processes, collective efficacy, and shared identity can be leveraged to support sustained engagement and action.^122^

### Strengths and Limitations

Several limitations to this study should be noted. The cross-sectional design precludes any inference of causality, and relationships among perceptions, worry, impairment, distress, and social factors may be bidirectional or dynamically evolving over time. Our reliance on self-reported data may also introduce bias due to recall or the limitations of current climate emotion scales. The self-reported nature of data collection may also contribute to shared method variance and affect responses to the pro-environmental behaviour scale, with participants potentially experiencing social desirability bias that artificially inflates scores.^123^ The sample, although national in scope, was recruited through online and MHCCA-affiliated channels, which may have led to an overrepresentation of individuals already concerned about climate change or engaged in related communities, thereby limiting generalizability. Because of this sampling bias, the sampling frame may inflate the relationships between the study variables, including perceptions, worry, impairment, and distress. The relatively homogeneous sample may also account for some of the substantial variance, as indicated by the high R^2 value, observed in the combined model. The relatively low explanatory power of pro-environmental behaviour, as well as the lack of a robust direct link from resilience to action, highlight important gaps in understanding which interventions may be useful for improving people’s lives. In particular, our models do not fully capture the social and contextual factors—such as group norms, leadership, cultural variations or opportunities for collective action—that may shape climate behaviour in real-world contexts. Given these complexities, we hope that our analyses can serve as a motivation for supporting larger, longitudinal, or experimental studies, which, although more costly investments, can better capture the mechanisms and directions of effect. Our goal in this study was not to provide a causal analysis, but to support the community-grounded theories of change that motivate many interventions aimed at addressing the adverse psychological and emotional consequences of climate change.

Future research should address the presented limitations by employing longitudinal and experimental designs, integrating objective behavioural and ecological data, and exploring group-based mechanisms for pro-environmental behaviour change. Studies that investigate the conditions under which distress motivates versus inhibits action, as well as the processes through which social support and resilience translate into collective efficacy, are particularly warranted. There is also a need for intervention research that rigorously tests and compares the effectiveness of climate cafés, peer support programs, citizen assemblies and other group-based approaches in reducing distress and promoting adaptive action. Future research could also analyze alternative pathways. For example, a future study could investigate whether social connection acts as a moderator between impairment and climate action, given that previous studies show that a strong social network can buffer against functional impairment.^124^

## Conclusion

This study advances understanding of the psychological and social processes that underlie climate-related distress and adaptation. Our findings underscore the importance of addressing both the cognitive and emotional impacts of climate change, as well as the supporting social connections and psychological resources that enable individuals and communities to adapt, act, and thrive. Effective responses to the mental health impacts of climate change must be multifaceted and contextualized, integrating accurate risk communication, emotional support, resilience-building, and opportunities for meaningful engagement and collective action. Through such efforts, we can better meet the urgent need to support mental health and well-being in an era of profound ecological transformation.

## Supplemental Material


Supplemental Material - Disrupting the Climate Distress Cascade: Testing Community-Grounded Theories of Change Using Secondary Data From a Cross-Sectional Canadian Study
Supplemental Material for Disrupting the Climate Distress Cascade: Testing Community-Grounded Theories of Change Using Secondary Data From a Cross-Sectional Canadian Study by Aayush Sharma, Tim K. Takaro, Carmen H. Logie, Maliha Ibrahim and Kiffer G. Card in Chronic Stress.

## Data Availability

Data is available at https://mhcca.ca/datasets.
