# Supplementary material for: Disrupting the Climate Distress Cascade: Testing Community-Grounded Theories of Change Using Secondary Data From a Cross-Sectional Canadian Study
Source: Chronic Stress (Thousand Oaks). 2026 Aug 28;10:24705470261472655. doi: 10.1177/24705470261472655 (PMC13527335; doi:10.1177/24705470261472655)
Supplement: Supplementary file 1 [file sj-pdf-1-css-10.1177_24705470261472655.pdf]

Supplementary Appendix

Supplement to: Disrupting the Climate Distress Cascade: Testing Community-Grounded Theories of Change Using Secondary Data from a Cross-Sectional Canadian Study

APPENDIX

Contents

**Item**	**Page Number**
**Appendix A:** Data Collection and Preparation: Study Scales.	**3**
**Appendix B:** Pearson Correlation Coefficients - CEDAR Scale.	**6**
**Appendix C:** Pearson Correlation Coefficients – All Variables.	**7**
**Appendix D:** Path Diagram for Model 1 (Climate Cascade).	**8**
**Appendix E:** Path Diagram for Model 2 (Social Mitigators).	**9**
**Appendix F.** Sensitivity Analysis for the Observed Social Network Variable.	**10**
**Appendix G.** Sensitivity Analysis Using Full Information Maximum Likelihood for Missing Data.	**12**
**Appendix H.** Unstandardized Covariances for the Final Combined Social–Climate Cascade Model.	**14**

Appendix A. Data Collection and Preparation: Study Scales**.**

Climate change perceptions: We used the Valkengoed Climate Change Perceptions Scale (CCPS) to assess participants' beliefs about the reality, human causation, and local and global impacts of climate change; items were recoded on a seven-point Likert scale from one (strongly disagree) to seven (strongly agree), with one item reverse-scored, and summed to yield a total score.[36] We used the single-item variation of the scale, which consisted of five questions measuring the five types of climate change perceptions. The five subscales consisted of questions regarding the reality of climate change, its causes, the perceived valence of consequences, spatial distance, and temporal distance. The Cronbach's alpha scores for the subscales were 0·98. 0·97, 0·90, 0·93, 0·89, respectively.[36] Higher scores indicate that the participant has a more pronounced perception that climate change is real, largely caused by human activities, will bring negative consequences, will affect their local area, and will be felt in the near future. Some examples of questions include "I believe that climate change is real," and "My local area will be influenced by climate change."

Climate change worry: We used the Climate Change Worry Scale (CCWS), which consists of ten items that reflect the frequency of worry or concern about climate change, scored from one (never) to five (always), and these were summed to create a total score.[37] The Cronbach's alpha score for the scale's internal consistency and unidimensionality is 0·95, indicating strong internal consistency. In another study, the same author assessed the test-retest reliability of the CCWS across two occasions, reporting Cronbach's alphas of 0·90 and 0·91, respectively.[37] Higher scores on the CCWS indicate greater worry about climate change. Some examples of questions include "I worry about climate change more than other people," and "I tend to worry when I hear about climate change, even when the effects of climate change may be some time away."

Climate change anxiety: We used the Clayton and Karazsia Climate Change Anxiety Scale (CCAS), a 13-item scale designed to assess levels of climate change anxiety as a psychological response to climate change.[5] The CCAS provided subscales for cognitive and functional impairment, with items recoded from one (never) to five (almost always); these subscales were then summed separately. Cognitive impairment is the subscale that shows how climate change affects mental capacity, including difficulty sleeping or rumination.[5,125] Functional impairment is the subscale that shows how climate change affects one's capacity in social and work situations.[5,125] These subscales were later used to define a latent impairment factor in model analyses. We used the 13-item CCAS, which has a Cronbach's alpha score of 0·92, showing strong internal consistency.[5,126] Larger scores indicate larger levels of anxiety as it relates to climate change. Some examples of questions include "Thinking about climate change makes it difficult for me to sleep," which is an example of cognitive impairment, and "My concerns about climate change undermine my ability to work to my potential," which is an example of functional impairment.

Climate perception and worry: We utilized the Six Americas Short SurveY (SASSY) to assess several aspects of climate change, including perceptions and worry.[38] The SASSY is a four-item measure scored from one (Extremely Important) to five (Not at all Important), with the total score summed. The study conducted a test-retest reliability analysis, achieving a Pearson Correlation coefficient of 0·66.[38] Higher scores indicate greater levels of importance and worry attributed to climate change. Examples of questions include "How important is the issue of climate change to you personally?" and "How much do you think climate change will harm you personally?"

Pro-environmental behaviour: Pro-environmental behaviour was assessed with the Larson Pro-Environmental Behaviour Scale (PEBS).[39] The PEBS is a 13-item scale that reflects the frequency of climate-friendly actions, scored from zero (never) to four (very often), and summed to produce a total score. The PEBS has Cronbach's alpha scores ranging from 0·64 to 0·84, indicating low to strong internal consistency.[39] Higher scores indicate a higher frequency of pro-environmental behaviours. Questions start with, "Thinking of your past behaviour, how frequently have you," and then provide examples of pro-environmental behaviours, such as "Recycled paper, plastic and metal," or "Conserved water or energy in my home."

Social belonging: We used the General Belongingness Scale (GBS), a 12-item scale that measures perceived social belonging, with six questions emphasizing inclusion and six focusing on exclusion.[40] This study recoded positively worded items from one (strongly disagree) to five (strongly agree), while negatively worded items were reverse-scored before all were summed. The GBS has a Cronbach's alpha score of 0·8, showing adequate internal consistency.[127] A higher score with the GBS scale indicates a higher level of belongingness. Some examples of questions include, "When I am with other people, I feel included," with five being strongly agree, and "I feel isolated from the rest of the world," with five being strongly disagree.

Social network: We used the Lubben Social Network Scale (LSNS-6) to capture the number of close relatives and friends in the participant's social network.[41] The scale has two sub-scales, the friends' subscale and the relatives' subscale. These two subscales were used to define a latent social network variable in the model analysis. Responses for the scale are scored from zero (none) to nine (nine or more) and summed to create subscale and overall scores. This study used the abbreviated six-item scale (LSNS-6), which has a Cronbach's alpha score of 0·83, showing strong internal consistency.[41] A higher score indicates lower levels of social isolation. Some examples of questions include, "How many relatives do you see or hear from at least once a month?" with five indicating you see five relatives that you see or hear from each month and "How many friends do you feel at ease with that you can talk about private matters?" etc.

Brief Resilience: We used the Brief Resilience Scale (BRS), a six-item scale that measures resilience as the ability to bounce back or recover from stress.[42] Responses from this scale are scored from one (strongly disagree) to five (strongly agree), with half the questions being positively worded and the other half being negatively worded; the negatively worded questions are recoded, allowing for a mean to be calculated. The Cronbach's alpha score for this scale ranges from 0·80 to 0·91, showing strong internal consistency.[42] A higher score indicates stronger levels of brief resilience. Some examples of questions include "I tend to bounce back quickly after hard times," which is worded positively, while "I have a hard time making it through stressful events," which is worded negatively.

Rugged Resilience: We used the Rugged Resilience Measure (RRM), a ten-item measure that examines internal resilience, or, in other words, psychological protective factors.[43] This study re-scored responses from one (strongly disagree or not at all) to five (strongly agree or a lot) and summed for total scores. Re-scoring was not required as all questions are worded positively. The scale showed strong internal consistency with a Cronbach's alpha score of 0·87.[43] Higher scores on this scale indicate higher levels of rugged resilience. Some examples of questions include, "I believe in myself" and "I can keep going despite difficulties." Both resilience measures (BRS and RRM) were used to define a latent factor for the model analysis.

Climate-Related Ecological Distress and Resilience: We used the Climate-Related Ecological Distress and Resilience Scale (CEDAR), a 12-item measure that examines both Climate-Related Ecological Distress and Resilience. This study uses a 5-point Likert Scale, from Never (one) to Always (five). When using the overall scale, distress items are reverse-coded. The scale has a Cronbach Alpha of 0.861 for the Distress Index and 0.769 for the Resilience Index. Higher scores indicate a higher level of climate-related ecological resilience. Some examples of questions include, “I feel sad over the current state of the environment,” or “I feel motivated to take individual action towards protecting the environment.”

Psychological distress: We used the Kessler Psychological Distress Scale (K10). The K10 comprises ten items scored from one (none of the time) to five (all of the time), which are summed to yield an overall distress score.[45,46] The K10 has a Cronbach's alpha of 0.93, showing high internal consistency.[46] Higher scores indicate higher levels of psychological distress and potential mental disorder. Some examples of questions include "During the last 30 days, about how often did you feel hopeless?" and "During the last 30 days, about how often did you feel worthless?"

Post-traumatic stress disorder: Post-traumatic stress disorder (PTSD) was assessed with the PTSD-8 scale.[47] The PTSD-8 is an eight-item measure to assess adverse psychological responses, scored from one (not at all) to four (all the time), with the total summed to come up with a total score. The Cronbach's alpha for the scale ranged from 0·83 to 0·85 across three samples.[47] Higher scores indicate a stronger psychological response. Examples of questions include "Recurrent thoughts or memories of the event" and "Avoiding activities that remind you of the event."

Anxiety: Anxiety levels were measured using the Generalized Anxiety Disorder measure (GAD-7).[48] The GAD-7 is a seven-item short-form measure to assess generalized anxiety, scored from zero (not at all) to three (nearly every day), with the scores summed to obtain a total score. This study re-scored the scale to be consistent with other scales in the study, ranging from one to four, using the same measure. The Cronbach's alpha for the scale was 0·92, showing strong internal consistency.[48] Higher scores indicate higher levels of anxiety. The scale begins with the statement, "Over the last two weeks, how often have you been bothered by the following problems?" with the scale then including behaviours, feelings, or thoughts such as "Feeling nervous, anxious, or on edge" and "Being so restless that it is hard to sit still."

Depression: To assess depression severity in this study, we utilized the Patient Health Questionnaire (PHQ-9).[49] The PHQ-9 is a nine-item measure to assess depression severity, scored from zero (not at all) to three (nearly every day), with the scores summed to obtain a total. We re-scored this variable, ranging from one (not at all) to four (nearly

every day), to align it with other variables in our study. The Cronbach's alpha for the PHQ-9 was 0·839, showing strong internal consistency.[128] Higher scores indicate more severe forms of depression. Questions in this measure begin with, "Over the last two weeks, how often have you been bothered by the following problems?" The scale then includes feelings and behaviours such as "Little interest or pleasure in doing things" or "Poor appetite or overeating."

Appendix B. Pearson Correlation Coefficients - CEDAR Scale.

	1. LSNS-6 friends score	2. LSNS-6 relatives score	3. GBS	4. RRM	5. BRS	6. PEBS	7. K10	8. CCWS	9. CCPS	10. CCAS cognitive	11. CCAS functional
**CEDAR Overall Resilience Scale**	0·18***	0·22***	0·38***	0·45***	0·39***	0·19***	-0·49***	-0·42***	-0·28***	-0·41***	-0·38***

Note: r values shown. *p < 0·05, **p < 0·01, ***p < 0·001 (uncorrected). All p-values for significant correlations are < 0·05 unless otherwise noted.

Abbreviations: LSNS: Lubben Social Network Scale; GBS: General Belongingness Scale; RRM: Rugged Resilience Measure; BRS: Brief Resilience Scale; PEBS: Larson Pro-Environmental Behaviour Scale; K10: Kessler Psychological Distress Scale; CCWS: Climate Change Worry Scale; CCPS: Valkengoed Climate Change Perceptions Scale; CCAS: Clayton and Karazsia Climate Change Anxiety Scale.

CEDAR overall resilience was positively correlated with social support ($r = 0·24$, $p < 0·001$), belongingness ($r = 0·38$, $p < 0·001$), rugged resilience ($r = 0·45$, $p < 0·001$), and pro-environmental behaviour ($r = 0·19$, $p < 0·001$), and negatively correlated with symptoms of PTSD ($r = -0·33$, $p < 0·001$), anxiety ($r = -0·38$, $p < 0·001$), depression ($r = -0·42$, $p < 0·001$), psychological distress ($r = -0·49$, $p < 0·001$), climate change worry ($r = -0·42$, $p < 0·001$), and climate-related cognitive and functional impairment ($r = -0·41$, $p < 0·001$ and $r = -0·38$, $p < 0·001$, respectively).

Appendix C. Pearson Correlation Coefficients – All Variables.

Variable	1	2	3	4	5	6	7	8	9	10	11	12	13	14	15	16	17	18
**1. CEDAR Overall Resilience Scale**	—																	
**2. LSNS-6 (Overall)**	.24***	—																
**3. LSNS-6 (Friends)**	.18***	.87***	—															
**4. LSNS-6 (Relatives)**	.22***	.83***	.44***	—														
**5. GBS**	.38***	.64***	.55***	.54***	—													
**6. PTSD-8**	-.33***	-.25***	-.18***	-.25***	-.47***	—												
**7. RRM**	.45***	.41***	.35***	.33***	.62***	-.45***	—											
**8. BRS**	.39***	.39***	.32***	.34***	.58***	-.51***	.75***	—										
**9. PEBS**	.19***	.14***	.14***	.10*	.06	.06	.10*	.02	—									
**10. GAD-7**	-.38***	-.24***	-.19***	-.22***	-.46***	.65***	-.53***	-.53***	.09*	—								
**11. PHQ-9**	-.42***	-.32***	-.25***	-.29***	-.58***	.59***	-.61***	-.60***	.03	.82***	—							
**12. K10**	-.49***	-.27***	-.17***	-.28***	-.47***	.54***	-.57***	-.58***	.27***	.69***	.71***	—						
**13. CCWS**	-.42***	-.12**	-.06	-.15***	-.28***	.35***	-.35***	-.38***	.45***	.53***	.46***	.78***	—					
**14. SASSY**	-.35***	-.16***	-.09*	-.19***	-.26***	.31***	-.35***	-.38***	.44***	.39***	.36***	.83***	.82***	—				
**15. CCPS**	-.28***	-.13**	-.06	-.17***	-.24***	.21***	-.33***	-.35***	.34***	.32***	.34***	.82***	.68***	.87***	—			
**16. CCAS (Overall)**	-.42***	-.18***	-.10*	-.20***	-.35***	.48***	-.37***	-.39***	.39***	.56***	.52***	.66***	.76***	.57***	.42***	—		
**17. CCAS (Cognitive)**	-.41***	-.18***	-.11**	-.20***	-.33***	.50***	-.37***	-.39***	.37***	.57***	.52***	.66***	.74***	.56***	.41***	.97***	—	
**18. CCAS (Functional)**	-.38***	-.15***	-.07	-.19***	-.33***	.39***	-.31***	-.33***	.37***	.47***	.45***	.59***	.70***	.52***	.38***	.92***	.79***	—

Note: r values shown. *p < 0·05, **p < 0·01, ***p < 0·001 (uncorrected).

Abbreviations: CEDAR: Climate-related Ecological Distress and Resilience Scale; LSNS: Lubben Social Network Scale; GBS: General Belongingness Scale; PTSD-8: Post-Traumatic Stress Disorder Scale; RRM: Rugged Resilience Measure; BRS: Brief Resilience Scale; PEBS: Larson Pro-Environmental Behaviour Scale; GAD-7: Generalized Anxiety Disorder Measure; PHQ-9: Patient Health Questionnaire; K10: Kessler Psychological Distress Scale; CCWS: Climate Change Worry Scale; SASSY: Six Americas Short SurveY; CCPS: Valkengoed Climate Change Perceptions Scale; CCAS: Clayton and Karazsia Climate Change Anxiety Scale.

Appendix D. Path Diagram for Model 1 **(**Climate Cascade).

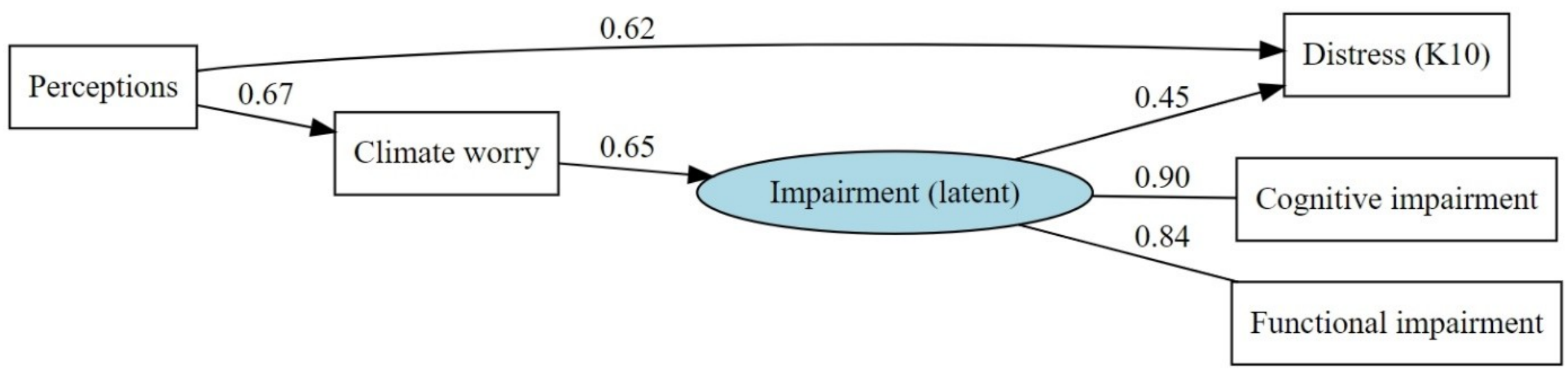

Note: Values shown are the standardized path coefficients (β) for the SEM analysis.

Appendix E. Path Diagram for Model 2 (Social Mitigators).

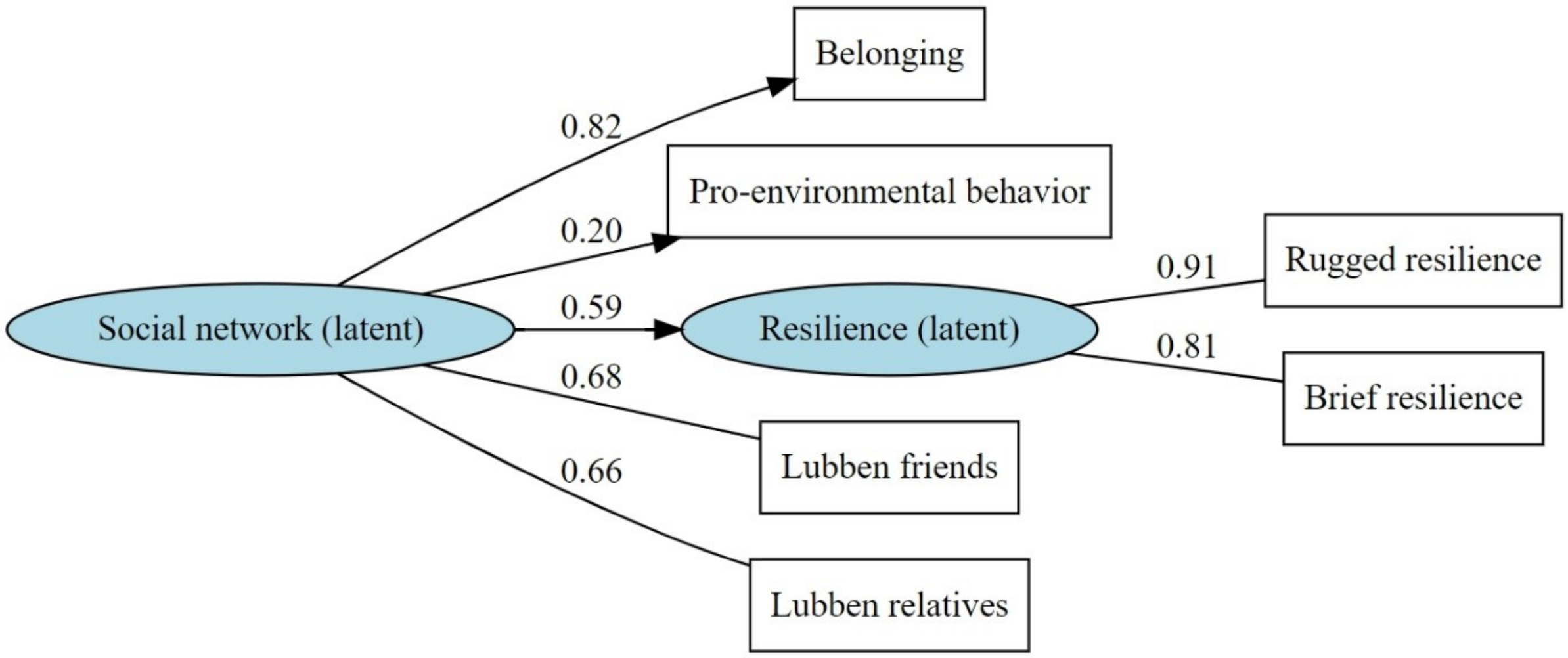

Note: Values shown are the standardized path coefficients (β) for the SEM analysis.

Appendix F. Sensitivity Analysis for the Observed Social Network Variable.

F.1. Standardized Path Coefficients for the Final Combined Social–Climate Cascade Model Using the Observed Total Social Network Variable.

Path	β (Std. All)	SE	z	p
Cascade Pathways				
Worry ← Perceptions	0.46	.06	13.53	<.001
Impairment ← Worry	0·93	.03	17·86	<.001
Impairment ← Perceptions	-0·21	.04	-4.52	<.001
Distress ← Impairment	0·32	.08	11.76	<.001
Distress ← Perceptions	0·55	.05	26·80	<.001
Social/Resilience Pathways				
Belonging ← Social network	0·66	.03	21·94	<.001
Resilience ← Social network	0·47	.03	10·73	<.001
Perceptions ← Resilience	–0·43	.05	–8·38	<.001
Worry ← Resilience	-0.28	.05	-7.78	<.001
Distress ← Resilience	–0·29	.07	–9·19	<.001
Distress ← Belonging	–0·06	.04	–2·62	.009
Action Pathways				
Perceptions ← Pro-env. action	0·38	.03	8·04	<.001
Worry ← Pro-env. action	0·33	·03	8·96	<.001

Note: N = 528.

F.2. Unstandardized Covariances for the Sensitivity Analysis Using the Observed Total Social Network Variable.

Covariances	Estimate	SE	z	p
Cognitive Impairment↔ Functional Impairment	0·35	0·81	0·43	.666
Impairment ↔ Social network	-6.42	2.05	-3.13	.002
Belonging ↔ Resilience	28.62	2.74	10.45	<.001
Pro-env. action ↔ Social network	21.10	6.21	3.40	0.001

Note: Covariance estimates are unstandardized covariances and not directly interpretable as correlations. Unstandardized measures are used as the variable types differ (observed vs latent) and would require different metrics to have clean correlations. The covariance between cognitive and functional impairment is non-significant; it was included to account for residual shared variance between subscales from the same measure.

F.3. Variance Explained in the Sensitivity Analysis Using the Observed Total Social Network Variable.

Scale	Estimate
RRM	0.790
BRS	0.701
CCAS Cognitive	0.817
CCAS Functional	0.725
CCPS	0.297
CCWS	0.584
K10	0.894
GBS	0.436
Resilience	0.219

Note: RRM = Rugged Resilience Measure; BRS = Brief Resilience Scale; CCAS = Climate Change Anxiety Scale; CCPS = Climate Change Perception Scale; CCWS = Climate Change Worry Scale; K10 = Kessler Psychological Distress Scale; GBS = General Belongingness Scale; Resilience is a Latent Factor of RRM and BRS; Impairment is a Latent Factor of CCAS Cognitive and CCAS Functional.

Appendix G. Sensitivity Analysis Using Full Information Maximum Likelihood for Missing Data.

G.1. Standardized Path Coefficients for the Final Combined Social–Climate Cascade Model Using Full Information Maximum Likelihood.

Path	β (Std. All)	SE	z	p
Cascade Pathways				
Worry ← Perceptions	0.47	.05	15.45	<.001
Impairment ← Worry	0·94	.02	23·60	<.001
Impairment ← Perceptions	-0·23	.03	-6.68	<.001
Distress ← Impairment	0·34	.06	14.95	<.001
Distress ← Perceptions	0·55	.04	32·58	<.001
Social/Resilience Pathways				
Belonging ← Social network	0·83	.15	12·04	<.001
Resilience ← Social network	0·59	.10	9·04	<.001
Perceptions ← Resilience	–0·42	.04	–10·03	<.001
Worry ← Resilience	-0.28	.05	-8.60	<.001
Distress ← Resilience	–0·27	.06	–10·34	<.001
Distress ← Belonging	–0·04	.03	–1·93	.053
Action Pathways				
Perceptions ← Pro-env. action	0·37	.02	10·49	<.001
Worry ← Pro-env. action	0·32	·03	9·94	<.001

Note: N = 737.

G.2. Unstandardized Covariances for the Sensitivity Analysis Using Full Information Maximum Likelihood.

Covariances	Estimate	SE	z	p
Cognitive Impairment↔ Functional Impairment	0·89	0·69	1·29	0.198
Impairment ↔ Social network	-4.23	0.85	-4.97	<.001
Belonging ↔ Resilience	14.90	3.37	4.42	<.001
Pro-env. action ↔ Social network	6.73	2.57	2.61	0.009

Note: Covariance estimates are unstandardized covariances and not directly interpretable as correlations. Unstandardized measures are used as the variable types differ (observed vs latent) and would require different metrics to have clean correlations. The covariance between cognitive and functional impairment is non-significant; it was included to account for residual shared variance between subscales from the same measure.

G.3. Variance Explained in the Sensitivity Analysis Using Full Information Maximum Likelihood.

Scale	Estimate
LSNS Friends	0.432
LSNS Relatives	0.434
RRM	0.779
BRS	0.719
CCAS Cognitive	0.809
CCAS Functional	0.703
CCPS	0.289
CCWS	0.589
K10	0.897
GBS	0.682
Resilience	0.349
Impairment	0.712

Note: LSNS = Lubben Social Network Scale; RRM = Rugged Resilience Measure; BRS = Brief Resilience Scale; CCAS = Climate Change Anxiety Scale; CCPS = Climate Change Perception Scale; CCWS = Climate Change Worry Scale; K10 = Kessler Psychological Distress Scale; GBS = General Belongingness Scale; Resilience is a Latent Factor of RRM and BRS; Impairment is a Latent Factor of CCAS Cognitive and CCAS Functional.

Appendix H. Unstandardized Covariances for the Final Combined Social–Climate Cascade Model.

Covariances	Estimate	SE	z	p
Cognitive Impairment↔ Functional Impairment	0·40	0·81	0·49	0.622
Impairment ↔ Social network	-4.53	0.97	-4.67	<.001
Belonging ↔ Resilience	13.17	3.82	3.44	0.001
Pro-env. action ↔ Social network	8.03	2.90	2.77	0.006

Note: N = 528. Covariance estimates are unstandardized covariances and not directly interpretable as correlations. Unstandardized measures are used as the variable types differ (observed vs latent) and would require different metrics to have clean correlations. The covariance between cognitive and functional impairment is non-significant; it was included to account for residual shared variance between subscales from the same measure.
